# Configuration Selection of the Multi-Loop Organic Rankine Cycle for Recovering Energy from a Single Source

**DOI:** 10.3390/e23111435

**Published:** 2021-10-30

**Authors:** Youyi Li, Tianhao Tang

**Affiliations:** The Institute of Power Drive and Control, Department of Electronic Engineering, Shanghai Maritime University, 1550 Haigang Ave., Shanghai 201306, China; thtang@shmtu.edu.cn

**Keywords:** Organic Rankine Cycle, configuration selection, number of loops selection, economic analysis, fluid selection, multi-objective optimization

## Abstract

The Organic Rankine Cycle (ORC) is a well-established way to recover energy from a single waste heat source. This paper aims to select the suitable configuration, number of loops, and working fluids for the Multi-Loop ORC (MLORC) by using multi-objective optimization. The thermodynamic and economic performance of MLORC in three various configurations was analyzed. Multi-objective optimizations of the series and parallel MLORC using different working fluid groups were conducted to find the optimal configuration, number of loops, and working fluid combination. The analysis results show that the series–parallel MLORC performed the worst among the three configurations. The optimization results reveal that series MLORC has a higher exergy efficiency than the parallel MLORC. The exergy efficiency of the optimal solution in series dual-loop, triple-loop, and quadruple-loop ORC is 9.3%, 7.98%, and 6.23% higher than that of parallel ORC, respectively. Furthermore, dual-loop is the optimal number of cycles for recovering energy from a single heat source, according to the grey relational grade. Finally, the series dual-loop ORC using cyclohexane\cyclohexane was the suitable configuration for utilizing a single waste heat source. The exergy efficiency and levelized cost of electricity of the series dual-loop ORC with the optimal parameters are 62.18% and 0.1509 $/kWh, respectively.

## 1. Introduction

Reducing ${\mathrm{CO}}_{2}$ to net zero is the most challenging task that humanity has ever encountered [1]. Low-grade heat power generation technologies are pivotal to achieve the target [2]. The technologies could recover industrial waste heat and improve fossil fuel utilization efficiency [3]. More than that, these could also be applied to some renewable energy that has low-grade heat [4]. The Organic Rankine Cycle (ORC) may have the most potential among all the low-grade heat power generation technologies [5,6]. Consequently, many researchers have focused on improving the thermodynamic and economic performance of the ORC [7] in the past decade.

One of the most effective ways to enhance the performance of the ORC is by increasing the number of loops of the ORC. Dual-loop ORC (DLORC) has shown great potential in thermodynamic and economic performance [8]. Furthermore, DLORC has a low carbon footprint [9], and high ${\mathrm{CO}}_{2}$ emission reduction [10]. Ouyang et al. [11] combined DLORC with marine diesel engine to make the ship meet the emission requirements. Ping et al. [12] investigated the environmental impact of DLORC in the application of CNG waste heat recovery, and the optimization results showed that DLORC emits only 43.74 tons of equivalent ${\mathrm{CO}}_{2}$. Boyaghchi and Chavoshi [13] studied the environmental impact rates of DLORC which was driven by solar energy. The analysis resutls revealed that the lowest environmental impact rates of the DLORC appeared in April. There are three main types of DLORC so far. The first one was proposed by Kusuda et al. [14], we call it a parallel type. In this type, the DLORC contained two basic ORCs. Each cycle in the parallel DLORC recovers part of the waste heat energy and no heat exchange occurs between them. The experimental results in article [14] show that parallel DLORC has a higher power output than a single loop ORC for Ocean Thermal Energy Conversion. Sciubba et al. [15] conducted a thermodynamic analysis on parallel DLORC. The analysis results showed that using two basic ORCs for a single source could significantly improve the thermal efficiency and heat source utilization rate. Sciubba et al. [15] also conducted fluid selection for the parallel DLORC. The results showed that R245fa had a better thermodynamic performance compared to R600. The second one was shown in the article [10]; we call it a series–parallel type. The waste heat was divided into two parts in the series–parallel configuration and absorbed by the two cycles. The two loops exchanged heat via an intermediate heat exchanger. Xia et al. [10] carried out the working fluid selection for the series–parallel DLORC by using multi-objective optimization. The objectives for the optimization are payback period, annual emission reduction, and exergy efficiency, and the decision method is improved grey relational analysis (GRA). The working selection results showed that cyclohexane/butane was the best suitable working fluids among 18 candidate working fluids pairs for the series–parallel configuration. The last type is a series one that is reported in the article [16]. In the series type, the waste heat is only absorbed by the first loop. The condenser in the first loop is the evaporator of the bottom cycle. Yuan et al. [16] compared the power output and exchange areas between the single ORC and series DLORC. The comparison results revealed that the power output of the series DLORC is 50% higher than that of the single ORC. However, the heat exchange areas are nearly the same. It can be seen from the above studies that increasing the number of loops could improve the performance of the ORC.

Furthermore, the concept of the triple loops was proposed to achieve a better performance [17]. In the article [17], two types of triple cycles were reported, one in series, another as a parallel type. The conclusions in the studies referenced showed that both types could enhance the performance of the system. Dwinanto [18] analyzed the thermodynamic performance of the triple cycle. The investigation showed that triple cycles had a higher energy utilization. Zhang et al. [19] proposed an innovative multi-loop ORC. The parametric analysis showed that Multi-Loop ORC (MLORC) could significantly improve the performance of the system.

The literature reviews showed that the dual-loop, triple-loop, and multi-loop ORC for recovering a single waste heat source showed better performance than that of a single ORC [10,14,15,16,19]. Moreover, the dual-loop cycle in three various types could significantly increase the performance of the ORC compared to a single cycle, according to the literature review. However, to our knowledge, no literature has compared the performance of the parallel, series–parallel, and series configurations. Therefore, selecting the well-suited configuration for the DLORC or Multi-Loop ORC was necessary. It is also worth selecting the appropriate working fluids and number of loops for the Multi-Loop ORC.

The aim of this paper is to select the suitable configuration, working fluid combinations, and number of loops for Multi-Loop ORC. The method used was analysis of the thermodynamic and economic performance of the Multi-Loop ORC (MLORC) in various configurations. The configurations are series, parallel, and series–parallel configurations. Furthermore, multi-objective optimization and multiple attribute decision making methods were also conducted to search for the best working fluid combination, the number of loops, and the configuration for the Multi-Loop ORC.

## 2. System Description

### 2.1. Exhaust Gas

In the present study, the waste heat source is reject from a low-speed two-stroke marine diesel engine. The waste heat source parameters are presented in Table 1. The exhaust gas’s composition is shown in Table 2. The composition parameters are applied for calculating the dew point and the thermodynamic properties.

### 2.2. Multi-Loop ORC

**Figure 1:**
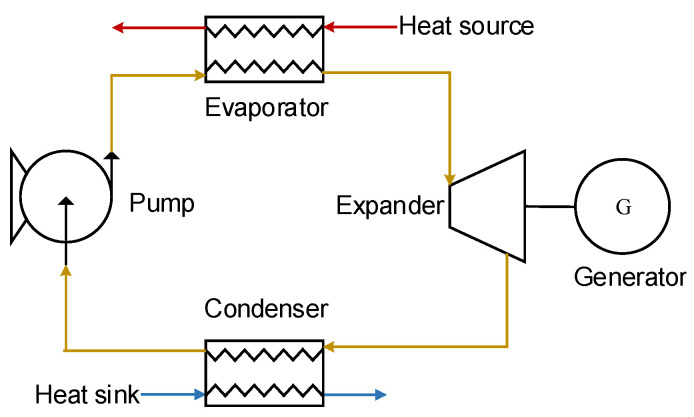
Basic Organic Rankine Cycle configuration.

ORC is considered the most prospective way to recover waste heat. A basic ORC consists of an evaporator, a condenser, an expander, and a working fluid pump [21], which is shown in Figure 1. The working fluid is heated into super-heated steam in the evaporator. Subsequently, the super-heated fluid expands in the expander to generate mechanic power and becomes low-pressure steam. The working medium rejects the heat into seawater through the condenser and becomes saturated liquid. Finally, the fluid coming from the condenser is circulated to the evaporator by the pump, and the whole cycle is completed. As can be seen in Figure 1, the basic ORC can be viewed as a single loop.

**Figure 2:**
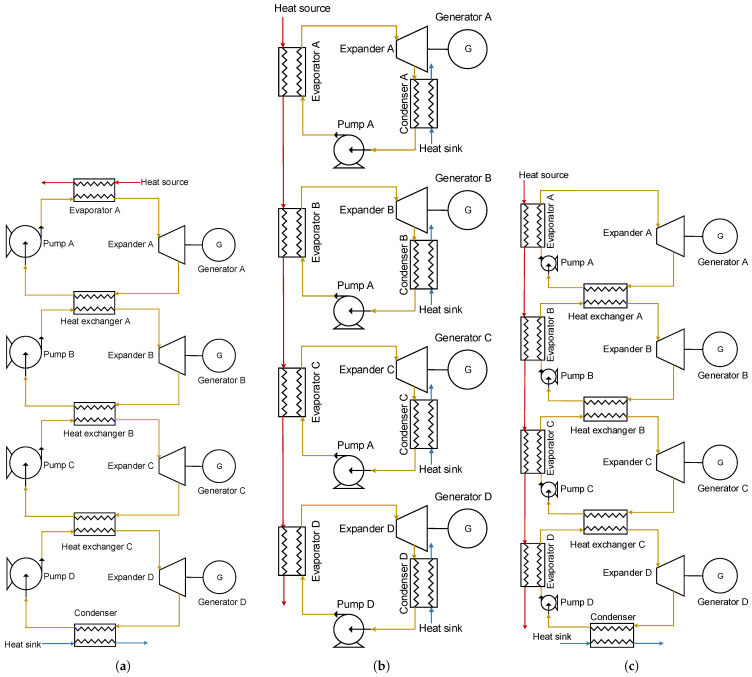
Three varying configurations of the Multi-Loop Organic Rankine Cycle. (**a**) Series type. (**b**) Parallel type. (**c**) Series–parallel type.

**Figure 3:**
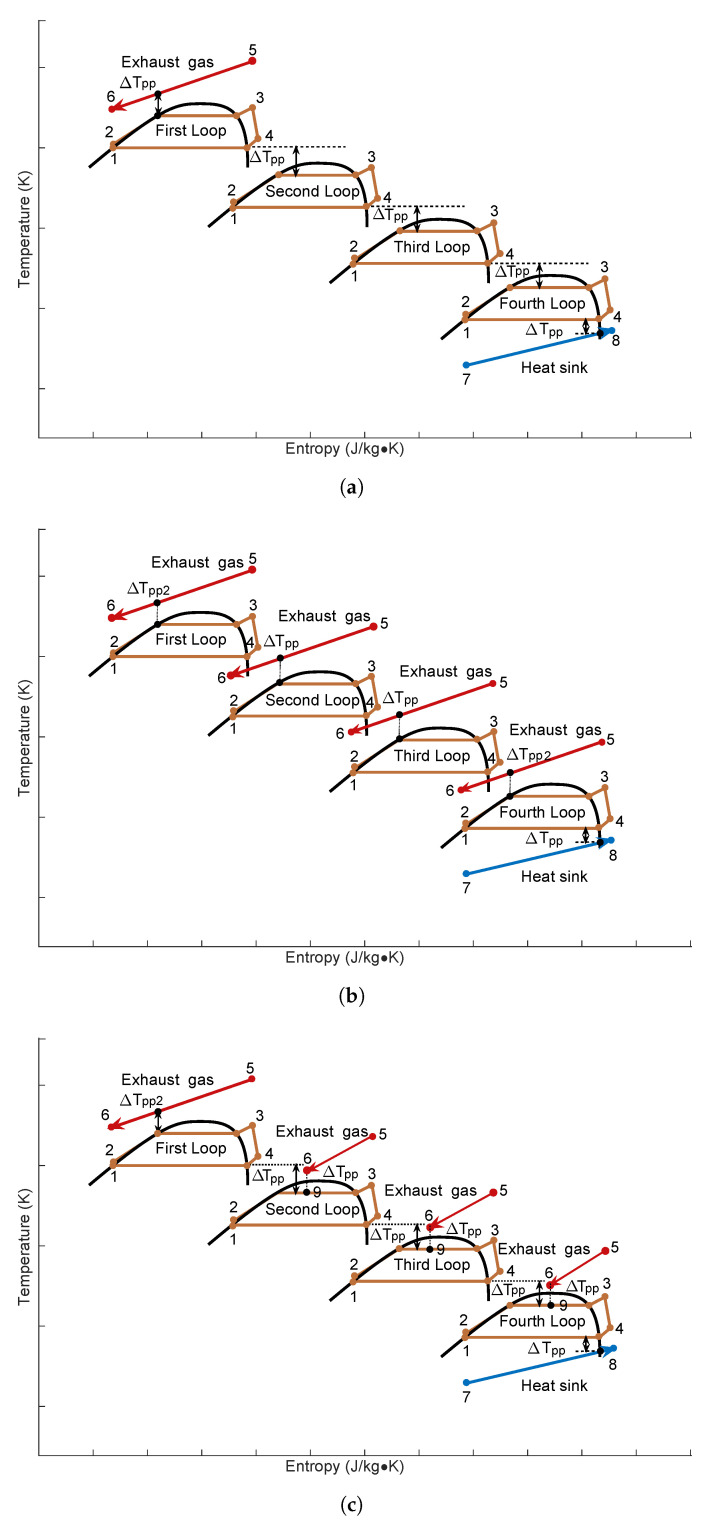
T-s diagrams of three various Multi-Loop Organic Rankine Cycle. (**a**) Series type. (**b**) Parallel type. (**c**) Series–parallel type.

Multi-Loop ORC (MLORC) means to have more than two loops in one Waste Heat Recovery System (WHRS). Figure 2 shows the Quadruple-Loop Organic Rankine Cycle with three various configurations. As can be seen in Figure 2a, series Quadruple-Loops ORC consists of one evaporator, four pumps, four turbines, three intermediate heat exchangers, and a condenser. It can be seen that there are four loops in the WHRS. In the first loop, the working fluid releases heat through heat exchanger A and completes the condensation procedure. The working fluid in the first cycle exchanges heat with the fluid in the second loop via the heat exchanger A. In the second loop, the upper heat exchanger A is used as an evaporator, and the working fluid changes into super-heated steam in heat exchanger A. The working fluid finishes the condensation process in lower heat exchanger B. The process in the third is the same as the process in the second loop. Finally, in the fourth loop, the working fluid rejects heat into the seawater through the condenser, and the whole cycle is finished. The T-s diagram of the Series Quadruple-Loop ORC is shown in Figure 3a. The cycle processes of series Dual-Loop ORC and series Triple-Loop ORC (TLORC) are similar to that of the series Quadruple-Loop ORC (QLORC) process.

Figure 2b illustrates the configuration of parallel Quadruple-Loop ORC. As can be seen, the exhaust gas is recovered by four basic ORCs in the parallel Quadruple-Loop ORC. This is because the basic ORC has differing performance at various heat source temperatures. There is an optimum fluid that will lead the WHRS to achieve the best performance. A portion of the heat in the exhaust gas is first recovered in the first loop, and the rest is utilized in the second, third, and fourth loops. In the parallel Dual-Loop ORC, the exhaust gas is recovered by two basic ORCs; in the Parallel Triple-Loops ORC, the exhaust gas is recovered by three basic ORCs. The T-s diagram of the parallel Quadruple-Loop ORC is shown in Figure 3b.

Figure 2c shows the configuration of series–parallel Quadruple-Loop ORC. The energy in the exhaust gas is divided into four parts and utilized by four evaporators. In the first loop, it is a basic ORC. The working fluid completes the condensed process in heat exchanger A. And the fluid rejects heat into the second loop via heat exchanger A. The first loop and the second loop are connected with heat exchanger A. In the second loop, heat exchanger A is used as a preheater. The working fluid from pump B is first preheated by the working fluid of the first loop. Then, the fluid carries out the evaporation process in evaporator B of the second cycle. The fluid changes into a super heated state. After this process, the super heated fluid enters expander B and converts the energy into mechanical power. The fluid that comes from expander B conducts a condensation process in heat exchanger B and is pumped to heat exchanger A by working fluid pump B. The process of the third loop is the same as the second loop. In the fourth loop, the working fluid that is circulated by fluid pump D first goes through heat exchanger C and is preheated by the third loop’s working fluid. The preheated working fluid is circulated into evaporator D and changes into super-heated steam. The steam expands in expander D and becomes low-pressure steam. The steam is condensed in the condenser and released the heat to the seawater. Finally, the sub-cooled liquid is sucked by pump D. Then the fluid is forced into heat exchanger D. The T-s diagram of the series–parallel Quadruple-Loop ORC is shown in Figure 3c.

### 2.3. Working Fluid Selection

The thermodynamic properties of the working fluid could significantly influence the power output of the MLORC [22]. Therefore, conducting working fluid selection for the MLORC is essential. Avoiding damage to the environment, the candidate working fluids should have zero ozone depletion potential value and low global warming potential. In addition, the principle of fluid selection is based on their critical temperature.The properties of the four candidate working fluids are presented in Table 3.

## 3. Materials and Methods

The selection of a suitable working fluid combination, configuration, and loop number for MLORC was found by analysis and optimization of the thermo-economic performance of the MLORC. Therefore, thermodynamic and economic models are needed.

### 3.1. Thermodynamic Model

The thermodynamic models of the MLORC are based on the first and second law of thermodynamics. The energy balance of the evaporators can be expressed as:

(1)Q˙ev,i=m˙r(h3,i−h2,i)(2)Q˙eg,i=m˙egcp,eg(Teg,in−Teg,out)

where $\dot{Q}$ is heat transfer rate, ${\dot{m}}_{r}$ is mass flow rate of the working fluid, *T* is temperature of the exhaust gas, *h* is specific enthalpy of the working fluid, $c_{p}$ is specific heat, subscript eg is exhaust gas, subscript in is inlet, subscript *i* is the sequence of the loop number, ev is the evaporator, and out is the outlet.

The energy balance of the heat exchangers for series and series–parallel MLORC can be described by

(3)Q˙he,i=m˙r,i(h4,i−h1,i)(4)=m˙r,i+1(h3,i−h2,i)

where the subscript he is the heat exchanger. The power output of the expanders are determined by

(5) $${\dot{W}}_{\mathrm{ex},i}={\dot{m}}_{r,i}\left(h_{3,i}-h_{4,i}\right)$$

where $W_{\mathrm{ex}}$ is the power out of the expander.

The power consumption of the working fluid pumps is calculated by

(6) $${\dot{W}}_{\mathrm{pu},i}=\frac{{\dot{m}}_{r,i}\left(h_{2\mathrm{is},i}-h_{1,i}\right)}{\eta_{\mathrm{pu},i}\eta_{\mathrm{pu},\mathrm{is},i}}$$

where $W_{\mathrm{pu}}$ is the power consumption of the pump, $\eta_{\mathrm{pu},\mathrm{is}}$ is isentropic efficiency of the pump, $\eta_{}$ is the efficiency of the pump, and subscript pu is the pump.

The energy balance of the condensers can be described by

(7)Q˙con=m˙r,i(h6,i−h1,i)(8)=m˙swcp,sw(Tsw,out−Tsw,in)

where con is the condenser and subscript sw is seawater.

The Net Power Output (NPO) of the MLORC can be deduced as:

(9) $${\dot{W}}_{\mathrm{NPO}}=\sum_{i=1}^{N}\left({\dot{W}}_{\mathrm{ex},i}-{\dot{W}}_{\mathrm{pu},i}\right)$$

where $W_{\mathrm{NPO}}$ is the net power output of the MLORC, and *N* is the total number of loops.

The thermal efficiency of the MLORC is calcualted as:

(10) $$\eta_{\mathrm{orc}}=\frac{{\dot{W}}_{\mathrm{NPO}}}{\sum_{i=1}^{N}{\dot{Q}}_{\mathrm{ev},i}}=\frac{{\dot{W}}_{\mathrm{NPO}}}{\sum_{i=1}^{N}{\dot{Q}}_{\mathrm{eg},i}}$$

where $\eta_{\mathrm{orc}}$ is the thermal efficiency of the MLORC.

Exergy is the maximum production possible and indicates the energy value of the system. The exergy of each state point in the MLORC can be obtained by

(11) $${\dot{E}}_{n}={\dot{m}}_{r}\left[\left(h_{n}-h_{0}\right)-T_{0}\left(s_{n}-s_{0}\right)\right]$$

where $E_{n}$ is the exergy of each state point, and $T_{0}$ is the ambient temperature.

The exergy loss in the evaporators and heat exchangers can be expressed as:

(12)I˙ev,se,1=E˙5,1−E˙6,1+E˙2,1−E˙3,1(13)I˙he,se,i=E˙4,i−1−E˙1,i−1+E˙2,i−E˙3,i(14)I˙ev,pa,i=E˙5,i−E˙6,i+E˙2,i−E˙3,i(15)I˙ev,sepa,1=E˙5,1−E˙6,1+E˙2,1−E˙3,1(16)I˙ev,sepa,i=E˙5,i−E˙6,i+E˙3,i−E˙9,i(17)I˙he,sepa,i=E˙4,i−1−E˙1,i−1+E˙2,i−E˙9,i

where *I* is the exergy loss in the equipment, subscript se is the series type, subscript pa is the parallel type, and sepa is the series–parallel type.

Likewise, the exergy losses of the other components are obtained by:

(18)I˙pu,i=E˙1,i−E˙2,i+W˙pu,i(19)I˙ex,i=E˙3,i−E˙4,i−W˙ex,i(20)I˙con,i=E˙4,i−E˙1,i−E˙7,i−E˙8,i

Based on the aforementioned calculation, the total exergy losses of the MLORC are calculated by

(21)I˙se,tot=I˙ev,se,1+∑i=1N(I˙ex,i+I˙pu,i)+∑i=2NI˙he,i+I˙con(22)I˙pa,tot=∑i=1N(I˙ex,i+I˙pu,i+I˙ev,i+I˙con,i)(23)I˙sepa,tot=∑i=1N(I˙ev,i+I˙ex,i+I˙pu,i)+∑i=2NI˙he,i+I˙con

where subscript tot is the total loss of the MLORC. Then, the exergy efficiency of the MLORC can be expressed as [26]

(24) $${\dot{\eta }}_{\mathrm{exer},y}=\frac{{\dot{W}}_{\mathrm{NPO},y}}{{\dot{I}}_{\mathrm{tot},y}+{\dot{W}}_{\mathrm{NPO},y}}$$

where the subscript *y* is the configuration type, and $\eta_{\mathrm{exer}}$ is the exergy effieciency of the MLORC.

**Figure 4:**
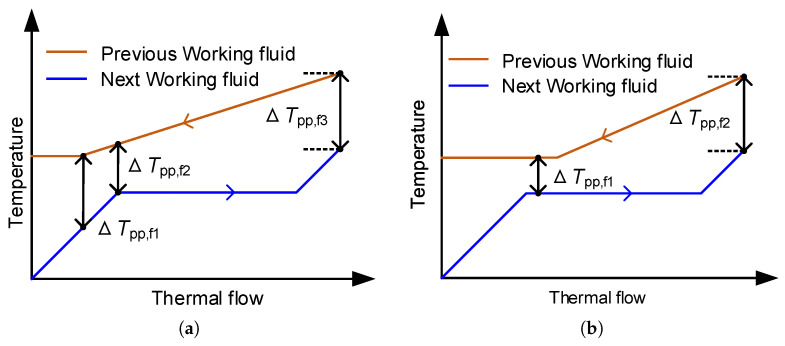

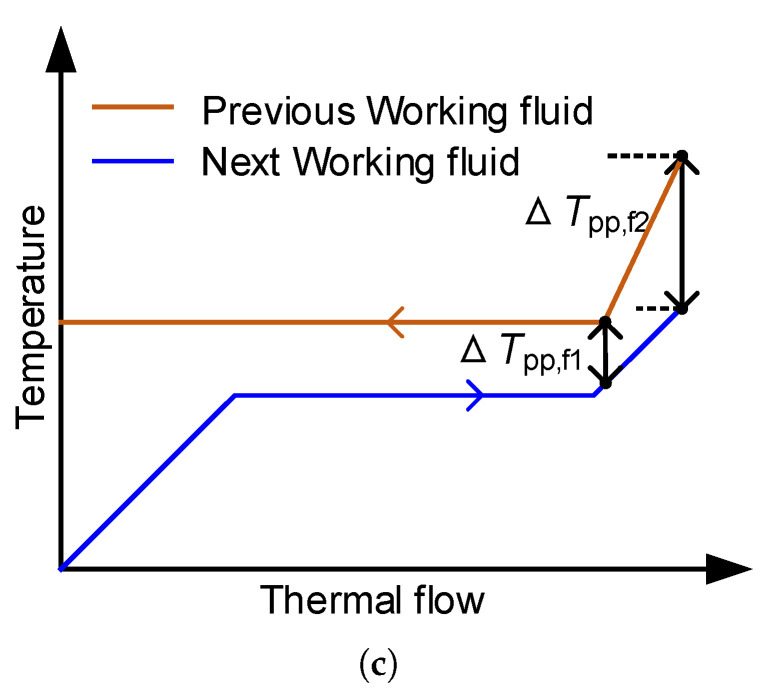
Probable circumstances of PPTD in series MLORC. (**a**) The pinch points may appear at the super-cooling zone, liquid saturation point, and the outlet of the next working fluid. (**b**) The pinch points may appear at the two-phase zone, and the outlet of the next working fluid. (**c**) The pinch points may appear at the super heated zone, and the outlet of the next working fluid.

**Figure 5:**
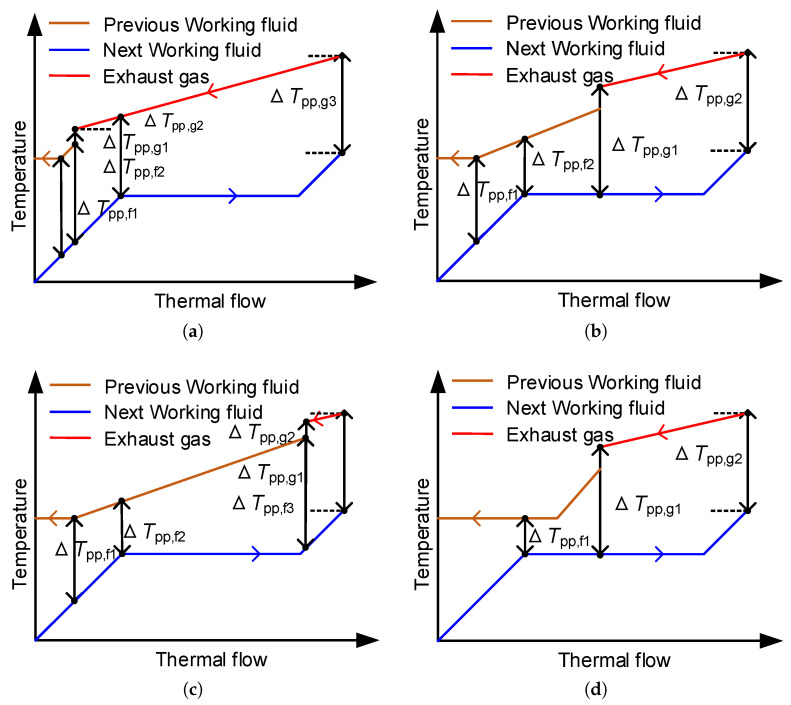

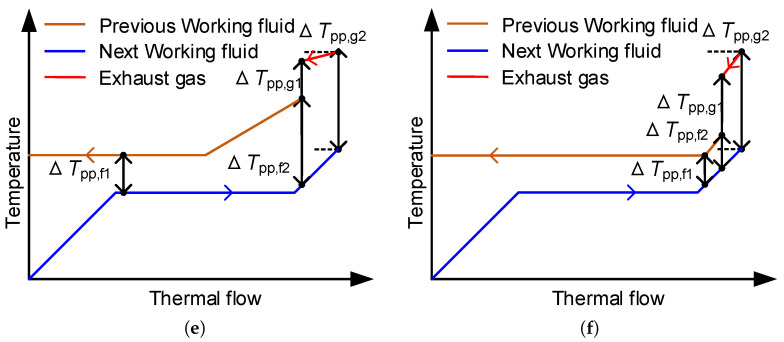
Probable circumstances of PPTD in series–parallel MLORC. (**a**) The next working fluid is preheated by the previous working fluid and exhaust gas, evaporated and superheated by the exhaust gas. (**b**) The next working fluid is preheated by the previous working fluid, evaporated by the previous working fluid and exhaust gas, and superheated by the exhaust gas. (**c**) The next working fluid is preheated and evaporated by the previous working fluid, and superheated by the previous working fluid and exhaust gas. (**d**) The next working fluid is preheated by the previous working fluid, evaporated by the previous working fluid and exhaust gas, and superheated by the exhaust gas. (**e**) The next working fluid is preheated and evaporated by the previous working fluid, and superheated by the previous working fluid and exhaust gas. (**f**) The next working fluid is preheated and evaporated by the previous working fluid, and superheated by the previous working fluid and exhaust gas.

The temperature difference at the pinch point has a significant influence on the heat exchanger performance and heat transfer. Therefore, the Pinch Point Temperature Difference (PPTD) is a constraint when performing thermodynamic analysis. According to the state of the working fluid in each heat exchanger, it can be divided into several circumstances during the calculation process. The probable circumstances of PPTD in series MLORC are shown in Figure 4, and the probable cases of PPTD in series–parallel MLORC are illustrated in Figure 5.

### 3.2. Economic Model

The economic model was used to evaluate the cost of the MLORC. For the heat exchangers, the cost has a direct relation with the heat exchange area. Therefore, we need to calculate the heat transfer area of the heat exchangers.

#### Heat Transfer Area

This paper used the Logarithmic Mean Temperature Difference (LMTD) method to calculate the heat transfer area. The LMTD method is a fundamental and general method used to calculate the heat transfer areas. Therefore, the heat transfer areas of each heat exchanger are calculated as:

(25) $$A=\frac{Q}{U\Delta T_{\mathrm{LM}}F}$$

where *A* is the heat transfer areas, *U* is the overall coefficient of heat transfer, *F* is the correction factor and is set as 0.95, and $\Delta T_{\mathrm{LM}}$ is given as in [23]

(26) $$\Delta T_{\mathrm{LM}}=\frac{\Delta T_{\max}-\Delta T_{\min}}{\ln(\Delta T_{\max}/\Delta T_{\min})}$$

where $\Delta T_{\max}$ is the maximum temperature difference in the heat exchanger, and $\Delta T_{\min}$ is the minimum temperature difference.

When the heat source for the next loop is the previous fluid, a plate type is selected to recover the waste heat. Then, $U_{\mathrm{pl}}$ of the plate heat exchanger is presented as [23]

(27) $$\frac{1}{U_{\mathrm{pl}}}=\frac{1}{\alpha_{r,h}}+\frac{1}{\alpha_{r,c}}+\frac{\delta }{k}$$

where α is the heat transfer coefficient, subscript h is the hot side, and subscript c is the cold side.

Moreover, when the heat source is exhaust gas, a shell and finned tube heat exchanger is applied. Then, $U_{\mathrm{st}}$ is calculated as follows [27]

(28) $$\frac{1}{U_{\mathrm{st}}}=\frac{1}{\alpha_{r,\mathrm{st},\mathrm{os}}}+\frac{d_{\mathrm{os}}}{\alpha_{s}d_{\mathrm{os}}}+\frac{d_{\mathrm{os}}\delta }{d_{\mathrm{ave}}k}+r$$

As can be seen in Equations (27) and (28), calculating *U* required estimation of the heat transfer coefficient of the hot side and cold side in the heat exchangers. In addition, there are three zones in the heat exchangers. Therefore, the heat transfer coefficient of each side and each zone of the heat exchanger needed to be calculated. Firstly, the heat transfer coefficient of the single phase working fluid in a plate heat exchanger is calculated as [28]

(29) $$Nu=0.724{\left(\frac{6\beta }{\pi }\right)}^{0.646}{Re}^{0.583}{Pr}^{1/3}$$

The boiling heat transfer coefficient of working fluid in a plate heat exchanger is expressed as [29]:

(30) $$\alpha_{\mathrm{tp},\mathrm{pl}}=1.926\frac{k_{r}}{D_{e,\mathrm{pl}}}Bo_{\mathrm{eq}}^{-0.3}Re_{\mathrm{eq}}^{0.5}Pr_{\mathrm{eq}}^{1/3}\left[\left(1-x\right)+x{\left(\frac{\rho_{l}}{\rho_{g}}\right)}^{0.5}\right]$$

The condensation heat transfer coefficient for the plate exchanger is deduced as [30]

(31) $$\alpha_{\mathrm{con},\mathrm{pl}}=4.118\frac{k_{r,l}}{D_{e,\mathrm{pl}}}Re_{\mathrm{eq}}^{0.4}Pr_{l}^{1/3}$$

The heat transfer coefficient on the hot side of the single-phase fluid in a plate heat exchanger is deduced by [29]

(32) $$\alpha_{h,\mathrm{pl}}=0.2121\frac{k_{r}}{D_{e,\mathrm{pl}}}Re_{r}^{0.78}Pr_{r}^{1/3}{\left(\frac{\mu_{r}}{\mu_{w,r}}\right)}^{0.14}$$

Secondly, the heat transfer coefficient of the single phase working fluid in the shell and tube exchanger is defined as [10]:

(33) $$\alpha_{r,\mathrm{st}}=0.023\frac{k_{r}}{D_{e,\mathrm{st}}}Re_{r}^{0.8}Pr_{r}^{a}$$

where *a* is 0.4 for heating and *a* is 0.3 for cooling. The boiling heat transfer coefficient of the working fluid is expressed as [31]:

(34) $$\alpha_{\mathrm{tp}}=\alpha_{l}\left[H_{1}H\;o^{H_{2}}{\left(25Fr_{\mathrm{lo}}\right)}^{H_{5}}+H_{3}Bo^{H_{4}}F_{fl}\right]$$

where

(35) $$\begin{array}{cc} \; & \alpha_{l}=0.023\frac{k_{r}}{D_{e,\mathrm{st}}}{\left(\frac{G_{r}\left(1-x\right)D_{e,\mathrm{st}}}{\mu_{r}}\right)}^{0.8}{\left(\frac{c_{p,l}\mu_{r}}{k_{r}}\right)}^{0.4} \end{array}$$

(36) $$\begin{array}{cc} \; & H\;o={\left(\frac{1-x}{x}\right)}^{0.8}{\left(\frac{\rho_{g}}{\rho_{l}}\right)}^{0.5} \end{array}$$

where, $F\;r_{l\;o}$ is a Froude number, $F_{fl}$ is a fluid-dependent parameter, and $H_{1}-H_{4}$ are dependent on the value of Ho and given in Table 4.

In the condenser, the mean heat transfer coefficient for film condensation in horizontal tubes is presented as follows [23]:

(37) $$\alpha_{\mathrm{con}}=0.943{\left[\frac{gk_{}^{3}\rho_{l}\left(\rho_{l}-\rho_{g}\right)\gamma }{D_{\mathrm{con}}\mu_{l}\left(T_{\mathrm{con}}-T_{w}\right)}\right]}^{1/4}$$

### 3.3. Equipment Cost Estimation

In this article, equipment module cost evaluation equations are applied to evaluate the total cost of the presented MLORC, including heat exchangers, expanders, working fluid pumps, and condensers. The cost evaluation equations are widely used in the cost estimation of the chemical equipment. The capital cost for each component of the MLORC is calculated as follows [32]:

(38) $$C_{\mathrm{BM},m}=C_{p,m}\left(B_{1,m}+B_{2,m}F_{M,m}F_{P,m}\right)$$

where $F_{P}$ is the pressure factor, $F_{M}$, $B_{1}$ and $B_{2}$ are constants and are shown in Table 5, and subscript *m* is the equipment type.

In Equation (38), $C_{p}$ can be expressed as follows [32]:

(39) $$\mathrm{lg}C_{p,m}=K_{1,m}+K_{2,m}\mathrm{lg}A_{m}+K_{3,m}{\left(\mathrm{lg}A_{m}\right)}^{2}$$

Furthermore the pressure factors, $F_{P}$, are given by the following expression [32]:

(40) $$\mathrm{lg}F_{P,m}=C_{1,m}+C_{2,m}\mathrm{lg}P_{m}+C_{3,m}{\left(\mathrm{lg}P_{m}\right)}^{2}$$

where $K_{1}$, $K_{2}$, $K_{3}$, $C_{1}$, $C_{2}$ and $C_{2}$ are empirical coefficients and are given in Table 5.

The cost of the equipment purchased in the year of 2020 can be deduced from the cost of year 2001 and estimated as

(41) $$C_{\mathrm{BM},m,2019}=C_{\mathrm{BM},m,2001}\cdot \frac{{\mathrm{CEPCI}}_{2019}}{{\mathrm{CEPCI}}_{2001}}$$

where the value of ${\mathrm{CEPCI}}_{2019}$ is 607.5 [10] and ${\mathrm{CEPCI}}_{2001}$ is 397.

Subsequently, the capital expenditure of a single loop can be calculated by

(42)Cse,i=CBM,he+CBM,ex+CBM,pu(43)Cpa,i=CBM,he+CBM,con+CBM,ex+CBM,pu(44)Csepa,i=CBM,he+CBM,eva+CBM,ex+CBM,pu

The total cost of the MLORC for each configuration is presented as

(45)Ctot,se=CBM,con+∑i=1NCse,i(46)Ctot,pa=∑i=1NCpa,i(47)Ctot,sepa=CBM,con+CBM,ev+CBM,ex+CBM,pu+∑i=2NCsepa,i

Finally, the levelized cost of energy (LCOE), which is an important metric of average cost of electricity over the lifetime of the system, can be evaluated by [23]

(48) $$L\;C\;O\;E=\frac{C_{\mathrm{tot}}\cdot C\;R\;F+C\;O\;M}{t_{\mathrm{ot}}\cdot {\dot{W}}_{\mathrm{NPO}}}$$

where

(49) $$C\;R\;F=\frac{j{\left(1+j\right)}^{LT}}{{\left(1+j\right)}^{LT}-1}$$

where LT is the life cycle time of MLORC and is set to 20, the discount rate *j* is 4.9% [23], COM is the cost of operations and maintenance and is assumed as 1.5% of $C_{\mathrm{tot}}$; $t_{ot}$ is the operational time per year and is set as 8000 h [24].

## 4. Multi-Objective Optimization Method

### 4.1. Optimization Algorithms Method

Multi-objective optimization is employed as a preferable approach to minimize or maximize multiple objective functions simultaneously [33]. Moreover, in these minimizations or maximizations, optimal decisions need to be made in the presence of trade-offs between two or more conflicting objectives. In the present article, the Non-dominated Sorting Genetic Algorithm II (NSGA II) [34], which has high computational efficiency, is applied to solve the multi-objective optimization problem by providing a Pareto Frontier set. The critical tuning parameters of NSGA II that were used in the present article are listed in Table 6. Furthermore, in order to improve the global searching ability, the population size is set as 100, and the maximum iterations are set as 100.

### 4.2. Multiple Attribute Decision Method

In the multiple attribute decision making (MADM) process on the Pareto Frontier, the Technique for Order Preference by Similarity to Ideal Solution (TOPSIS) [36] is applied to obtain the Pareto optimal solution. The TOPSIS calculation steps are as follows:

Step 1: find the positive ideal solutions $Z^{+}$ and negative ideal solutions $Z^{-}$ which can be described as:

(50)Z+=(maxz11,z21,⋯,zn1,maxz12,z22,⋯,zn2)(51)=(Z1+,Z2+)(52)Z−=(minz11,z21,⋯,zn1,minz12,z22,⋯,zn2)(53)=(Z1−,Z2−)

where *z* is the objective value of the individual.

Step 2: calculate the Euclidean distances *D* of each individual in the Pareto Frontier:

(54) $$\begin{array}{c} D_{i}^{+}=\sqrt{\sum_{j=1}^{m}{\left(Z_{j}^{+}-z_{ij}\right)}^{2}} \end{array}$$

(55) $$\begin{array}{c} D_{i}^{-}=\sqrt{\sum_{j=1}^{m}{\left(Z_{j}^{-}-z_{ij}\right)}^{2}} \end{array}$$

Step 3: calculate the relative closeness $\xi_{i}$ of each individual in the Pareto Frontier as follows:

(56) $$\xi_{i}=\frac{D_{i}^{-}}{D_{i}^{+}+D_{i}^{-}}$$

Step 4: rank all the individuals in the Pareto Frontier according to the relative closeness $\xi_{i}$; the individual that has the highest value $\xi_{i}$ is set as the optimal solution on the Pareto Frontier.

After all the optimal solutions of the Pareto Frontiers have been calculated, grey relational analysis (GRA) is used to evaluate the solutions. The GRA could combine the entire range of performance attribute values into a single value [37]. Consequently, all the optimal solutions with multiple attributes could be compared easily using a GRA method. The process of GRA can be calculated as the following steps:

Step 1: Calculate the relational coefficient $\zeta_{i}$ of each optimal solution as follows:

(57) $$\zeta_{ij}=\frac{\Delta \min+\Omega \Delta \max}{\Delta_{ij}+\Omega \Delta \max}$$

where

(58)Δij= x0j−xij(59)Δmin=minΔij,i=1,2,…,m;j=1,2,…n(60)Δmax=maxΔij,i=1,2,…,m;j=1,2,…n

Step 2: Calculate the grey relational grade $R_{i}$ of each optimal solution as follows:

(61) $$R_{i}=\sum_{j=1}^{n}w_{j}\zeta_{ij}\left(i=1,2,\ldots ,m\right)$$

where, *w* is the weight coefficient, *w* is considered equal in this paper, *R* is the grey relational grade. After the GRA process, rank all the optimal solutions according to the grey relational grade *R*.

### 4.3. Objective Functions and Decision Variables

Thermodynamic and economic performance are the two most significant objectives in designing a waste heat recovery system. Therefore, LCOE and exergy efficiency are selected as the objectives of the present study.

The decision variables for Series MLORC are the evaporation temperature $T_{\mathrm{ev},1}$ of the first loop, the super-heat temperature $T_{\sup,1}$ of the first loop, the outlet temperature $T_{\mathrm{ex},\mathrm{out}}$ of the exhaust gas, the condensation temperature $T_{\mathrm{con},i}$ of each loop, the pinch point temperature $T_{\mathrm{pp},i}$ in the intermediate heat exchanger, and the pinch point temperature $T_{\mathrm{pp},\mathrm{con}}$ in the condenser of the last loop.

The decision variables for Parallel MLORC are the evaporation temperature $T_{\mathrm{ev},i}$ of each loop, the super-heat temperature $T_{\sup,i}$ of each loop, the exhaust gas outlet temperature $T_{\mathrm{ex},\mathrm{out},i}$ of each loop, the condensation temperature $T_{\mathrm{con},i}$ of each loop, the pinch point temperature $T_{\mathrm{pp},\mathrm{con},i}$ in the condenser of each loop, and the heat distribution of the exhaust gas.

### 4.4. Constraints

Constraints in the present multi-objective optimization are general constraints and boundaries on variables. Exhaust outlet temperature, which is one of the general constraints, should not drop below the dew point temperature to avoid low-temperature corrosion. The acid dew point of the exhaust gas is the temperature at which the gaseous acid will change into liquid acid at a given temperature. The liquid acid could cause corrosion of the evaporator, and affect the operation safety of the equipment [38]. In addition, the exhaust gas outlet temperature is considered as a variable. Therefore, the exhaust outlet temperature should not drop below the dew point in the optimization process. The dew point temperature is related to the partial pressure of ${\mathrm{SO}}_{3}$ and water vapor, and can be deduced from the following equation [39]:

(62) $$T_{\mathrm{dew}}=203.25+27.6\mathrm{lg}\left(P_{H_{2}O}\right)+10.83\mathrm{lg}\left(P_{{\mathrm{SO}}_{3}}\right)+1.06{\left(\mathrm{lg}(P_{{\mathrm{SO}}_{3}})+8\right)}^{2.19}$$

The PPTD in the liquid–liquid heat exchanger should satisfy Equation (63), and in the vapor–liquid heat exchanger should meet Equation (64).

(63)minTpp,f1,Tpp,f2,Tpp,f3 ≥ 5(64)minTpp,g1,Tpp,g2,Tpp,g3 ≥ 10

The basic parameters, constraints, and boundaries of the MLORC are presented in Table 7.

## 5. Results and Discussion

### 5.1. Model Validation

**Figure 6:**
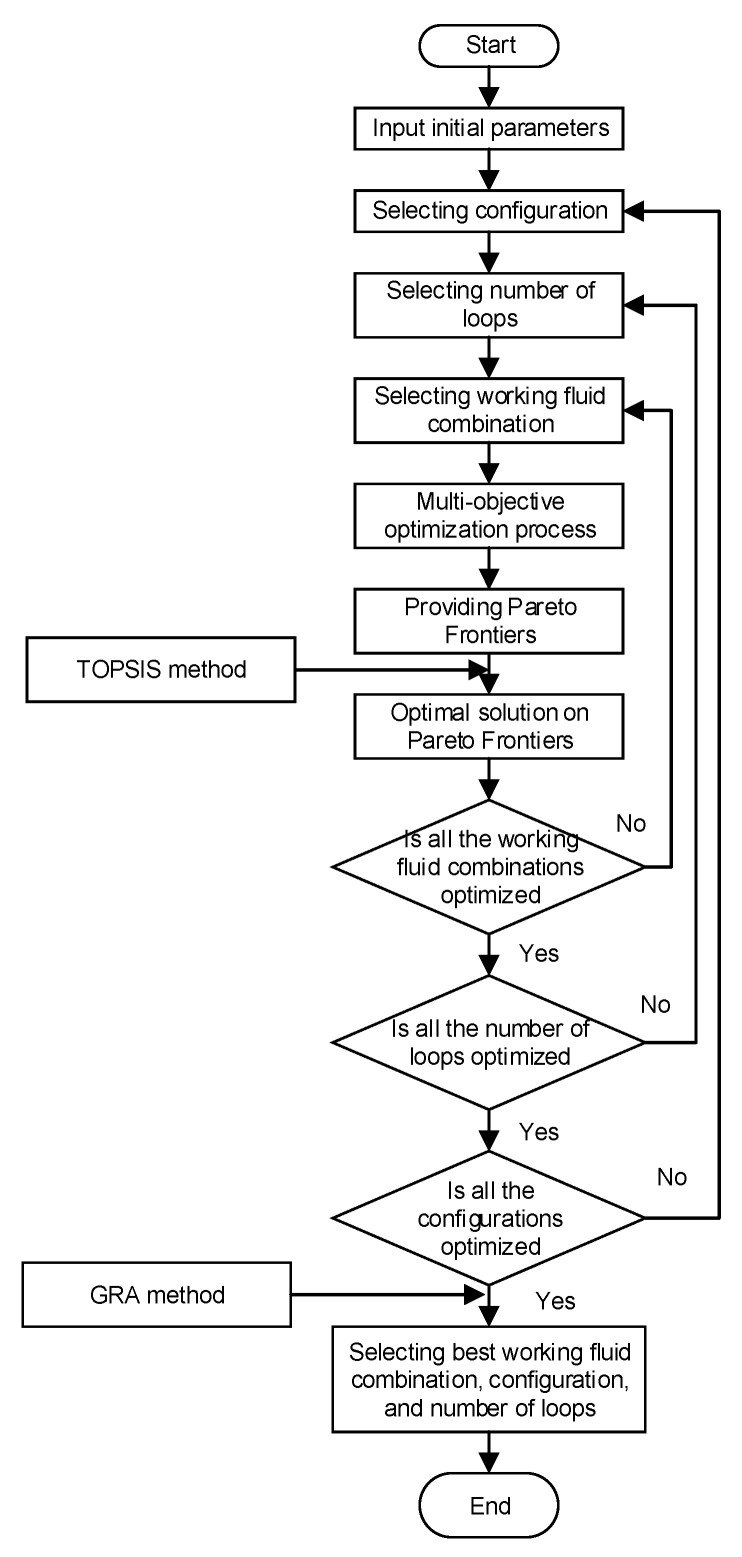
The flow chart of the implantation in MATLAB.

The thermodynamic model of MLORC is implemented in MATLAB 2016a with the CoolProp 6.41 [42]. The CoolProp software provides the thermodynamic properties of the working fluids and the exhaust gas. The flow chart of the implantation in MATLAB can be seen in Figure 6.

The thermodynamic model developed in MATLAB is validated with the results in Reference [43]. The comparison results between the present solution and the results in Reference [43] are shown in Table 8. The discrepancies between these two results may be due to the software that is used to calculate the thermodynamic and transport properties of the fluids. REFPROP software is applied in the article [43], while CoolProp software is used in the present study.

The economic model is validated with the results in the article [23]. The results of the present study and the results in Reference [23] are shown in Table 9. The discrepancies between these two results may be due to the difference in the method of calculating the heat transfer coefficient.

### 5.2. Series MLORC

In the series MLORC, there is only one evaporator for recovering energy from the exhaust. Therefore, the evaporation temperature of the first cycle is considered to be constant in the analysis. The variable is the evaporating temperature of the second loop. Additionally, R245fa is applied for all cycles due to its superior performance [44].

**Figure 7:**
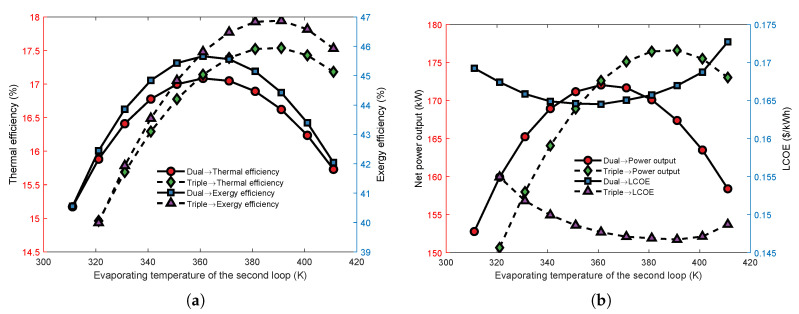
The effect of second loop’s evaporating temperature on the thermoeconomic performance of the series MLORC. (**a**) Effect on thermal and exergy efficiency. (**b**) Effect on power output and LCOE.

Figure 7 shows the effect of the second cycle’s evaporating temperature on the thermoeconomic performance of the series MLORC. As the evaporating temperature rises, the thermodynamic indicators of the series MLORC first rise and then decrease. However, the economic indicator LCOE has an opposite trend to NPO. The results indicated that in series dual-loop ORC, there is an optimal evaporation temperature at which the whole cycle could achieve the best performance. Moreover, the evaporation temperature at which the TLORC’s maximum power output occurs is higher than for the dual-loop ORC. Moreover, the maximum thermal efficiency and exergy efficiency of the TLORC is higher than that of the dual-loop ORC. The results revealed that the series MLORC might have a better thermoeconomic performance for recovering energy from a single source by increasing the loop number.

**Figure 8:**
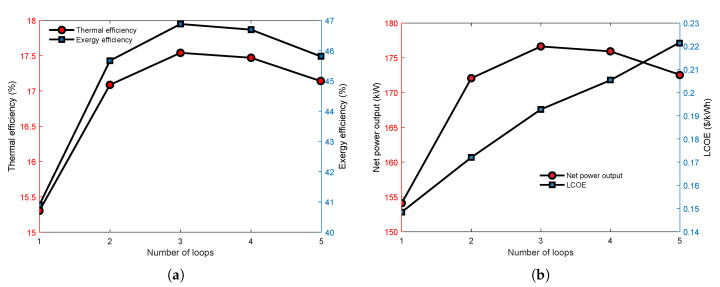
The effect of loop number on the thermoeconomic performance with the series MLORC. (**a**) The effect on thermal and exergy efficiency. (**b**) The effect on power output and LCOE.

Figure 8 presents that the effect of loop number on the thermoeconomic performance of the series MLORC. In the analysis, the working fluid is R245fa for each cycle, and the evaporating temperature of the first cycle is considered constant. Therefore, the temperature difference between evaporating temperature and condensation temperature in each cycle is considered equal. As can be seen, as the number of loops increases, the thermodynamic indicators first rise and then decrease. However, an increasing number of loops causes the increase in LCOE. The phenomenons are consistent with the results in Figure 7. The turning point for deteriorating performance is the third cycle.

Furthermore, the TLORC has the best performance in thermal efficiency, exergy efficiency, and NPO. These results illustrated that there is an optimal number of cycles for series configuration with which the system can achieve the best performance. Additionally, when the number of cycles is above two and below five, the thermodynamic performance is better than a single cycle. However, the economic performance becomes worse as the number of cycles increases.

### 5.3. Parallel Multi-Loop ORC

In the parallel MLORC, the energy of the exhaust gas is divided into several parts, and a basic ORC recovers each part. In the analysis, in order to study the effect of the number of cycles, the restriction of the dew point temperature was removed. Moreover, the working fluids for all the basic ORCs are R245fa.

**Figure 9:**
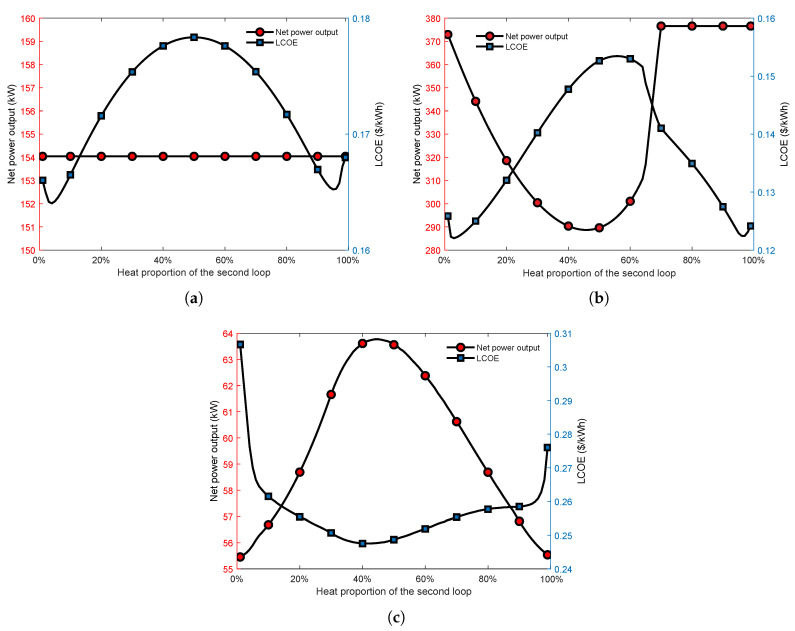
Effect of the distribution of heat energy on the thermoeconomic parallel dual-loop ORC under various outlet temperatures and inlet temperatures of the exhaust gas. (**a**) Inlet and outlet temperature of the heat source are higher than the working fluid critical temperature. (**b**) $T_{\mathrm{eg},\mathrm{in}}=537.75\;K,T_{\mathrm{eg},\mathrm{out}}=350\;K$. (**c**) $T_{\mathrm{eg},\mathrm{in}}=400\;K,T_{\mathrm{eg},\mathrm{out}}=320\;K$.

Figure 9 presents that the effect of heat proportion of the second cycle on the thermodynamic and economic performance of the parallel dual-loop ORC. It is viewed that in Figure 9a, when the inlet and outlet temperature of the exhaust gas is higher than the working fluid critical temperature, increasing the heat proportion of the second loop does not affect the thermodynamic performance of the parallel MLORC. A possible reason may be that there is no pinch point limit in this condition. Furthermore, the LCOE has a smaller drop at first. The reason could be that the decrease in the energy distribution of the first ORC allows an increase in the exhaust gas exit temperature of the first ORC. This results in an increase in the LMTD and thus a reduction in the heat exchange area. When the heat proportion of the second ORC increases from 4% to 50%, the LCOE rises. When the heat proportion of the second ORC increases from 50% to 96%, the LCOE decreases. The reason for this phenomenon may be due to the change in LMTD.

**Figure 10:**
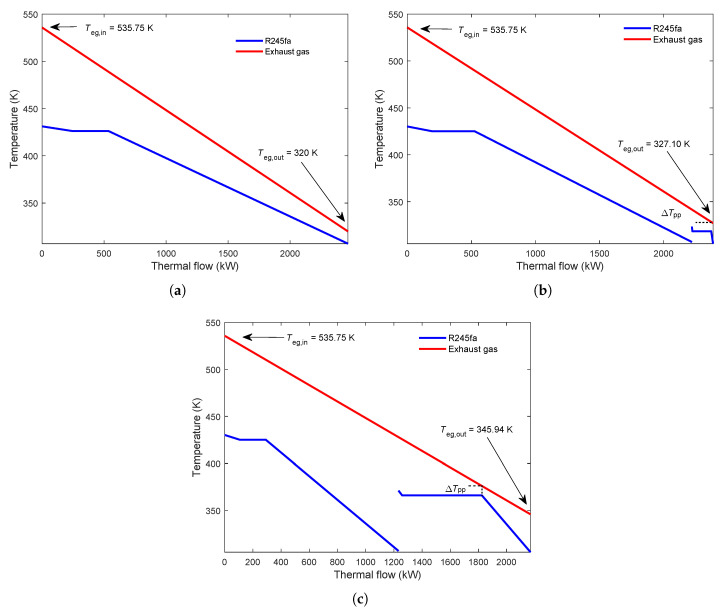
Composite Curves for single loop ORC and dual-loop ORC in the scenario $T_{\mathrm{eg},\mathrm{in}}=537.75\;K,T_{\mathrm{eg},\mathrm{out}}=320\;K$. (**a**) Single loop ORC. (**b**) Heat proportion of the second cycle is 10%. (**c**) Heat proportion of the second cycle is 50%.

As can be seen in Figure 9b, in the scenario $T_{\mathrm{eg},\mathrm{in}}=537.75\;K,T_{\mathrm{eg},\mathrm{out}}=350\;K$, increasing the heat distribution of the second cycle leads to a decrease in power output. Then, after reaching a minimum value, the power increases when increasing the proportion of energy. Eventually, increasing heat distribution, the parallel dual-loop ORC’s power output is equal to that of a single basic ORC. The reason for the results can be seen in Figure 10. It can be seen that when the heat proportion of the second cycle is 10%, the exhaust gas outlet temperature is 327.10 K. Furthermore, when the heat proportion of the second cycle is 50%, the exhaust gas outlet temperature is 345.94 K. The phenomenons mean that the exhaust gas is not fully utilized. Therefore the power output is lower than that of a single ORC.

**Figure 11:**
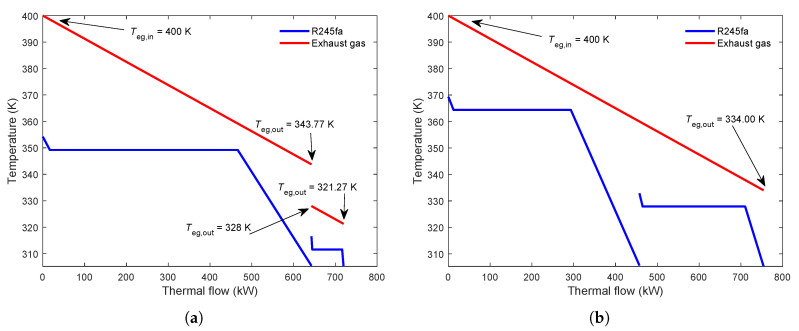
Composite Curves for dual-loop ORC in the scenario $T_{\mathrm{eg},\mathrm{in}}=400\;K,T_{\mathrm{eg},\mathrm{out}}=320\;K$. (**a**) Heat proportion of the second cycle is 90%. (**b**) Heat proportion of the second cycle is 50%.

Figure 9c presents that, when the heat proportion of the second cycle increases, in the condition $T_{\mathrm{eg},\mathrm{in}}=400\;K,T_{\mathrm{eg},\mathrm{out}}=320\;K$, the NPO first rises and then decreases. Therefore, the increase in NPO leads to a decrease in LCOE. The reason for this result can be seen in Figure 11.

It can be observed that, when the heat proportion is 90% in the upper ORC, the exhaust gas has an outlet temperature of 343.77 K due to the PPTD limit. In the lower cycle, the exhaust gas outlet temperature is 321.27 K because of the PPTD restriction. It can be seen that a part of the energy is not recovered. The residual part accounts for 21.3% of the exhaust gas energy. It can be seen that, when the heat distribution is 50% in the first ORC, the exhaust gas could be fully utilized in the upper cycle. However, in the second cycle, the exit temperature of the exhaust gas is 334.0 K. The remaining energy represents 17.5% of the total exhaust energy that can be used. These results suggest that optimization in heat proportion of the exhaust gas energy could improve the thermodynamic and economic performance of the parallel MLORC.

**Figure 12:**
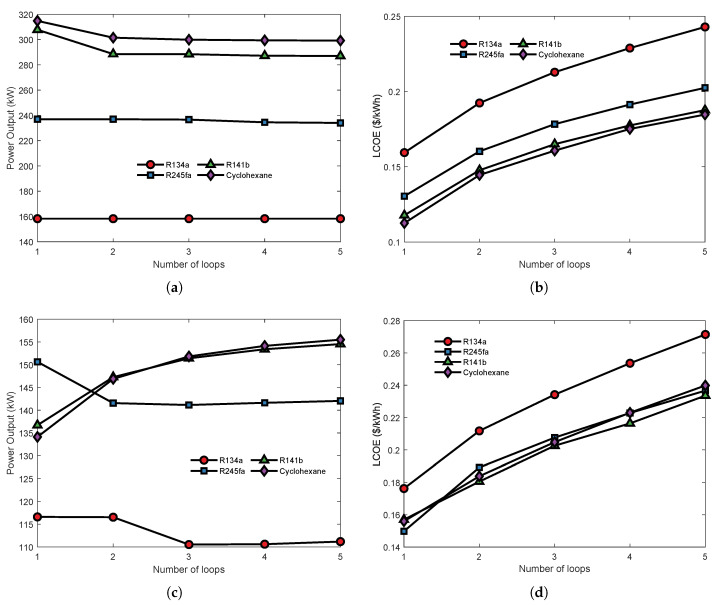
Effect of the second loop’s evaporating temperature on the thermoeconomic performance of the series MLORC. (**a**) Effect on power output under $T_{\mathrm{eg},\mathrm{in}}=537.75\;K,T_{\mathrm{eg},\mathrm{out}}=T_{\mathrm{dew}}$. (**b**) Effect on LCOE under $T_{\mathrm{eg},\mathrm{in}}=537.75\;K,T_{\mathrm{eg},\mathrm{out}}=T_{\mathrm{dew}}$. (**c**) Effect on power output under $T_{\mathrm{eg},\mathrm{in}}=450\;K,T_{\mathrm{eg},\mathrm{out}}=350\;K$. (**d**) Effect on LCOE under $T_{\mathrm{eg},\mathrm{in}}=450\;K,T_{\mathrm{eg},\mathrm{out}}=350\;K$.

Figure 12 shows that the influence of the number of cycles on the parallel MLORC power output and LCOE. As can be seen in Figure 12a, increasing the number of cycles does not affect the system’s power output when the working fluid is R134a or R245fa. However, when the working fluid is cyclohexane or R141b, the increase in the number of cycles causes a decrease in NPO. The decline stops when the number of loops is two. MLORCs with dual-loop and more than two cycles have equal power output. The parallel MLORC has the maximum NPO when using cyclohexane and the lowest NPO when using R134a. The results are in accord with the previous studies. R134a shows poor performance when the exhaust temperature is high. As can be seen in Figure 12b, it is obvious that the economic indicator LCOE rises with the increasing number of cycles. Similarly, the working fluid R134a performs the worst among all the four candidate working fluids.

Figure 12c,d present the effect of loop number on power output and LCOE of the parallel MLORC in the circumstance $T_{\mathrm{eg},\mathrm{in}}=450\;K,T_{\mathrm{eg},\mathrm{out}}=350\;K$. As can be seen, when the working fluid is R134a, the single ORC has the same NPO with dual-loop. However, the NPO of the TLORC is lower than that of single ORC or dual-loop ORC. Moreover, the MLORC that has a triple loop or more than triple loops and has an equal NPO. It can be seen that, when using R245fa as the system working fluid, the parallel with more than two cycles is lower than the NPO of a single loop ORC. Interestingly, when using cyclohexane or R141b as the working fluid, increasing the number of loops leads to an increase in the NPO of the parallel MLORC. A possible reason for this result may be due to the PPTD limit in the evaporator of each cycle. Figure 12b reports that increasing the number of loops causes an increase in the LCOE. However, it can be seen that R141b has the best economic performance when the number of cycles is more than two. These results suggest that, although increasing the number of cycles can increase power output, and it will worsen economic performance.

### 5.4. Series—Parallel Multi-Loop ORC

In this analysis, R245fa is used as the working fluid for each cycle.

**Figure 13:**
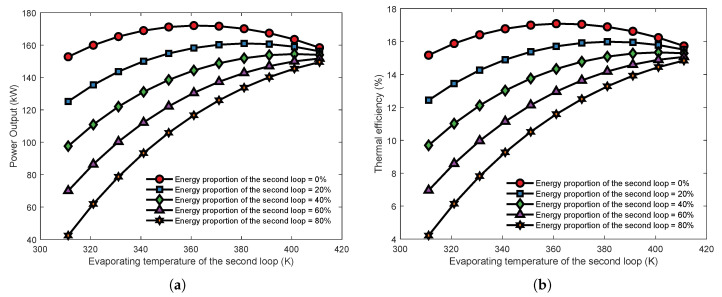
Effects of heat proportion and evaporating temperature of the second loop on power output and thermal efficiency with series–parallel configuration. (**a**) Power output. (**b**) Thermal efficiency.

Figure 13 shows that the increase in the evaporating temperature of the second loop causes an increase in power output and thermal efficiency when the proportion of the second loop is 40%, 60%, and 80%. There are some discrepancies between these results and that of the series dual-loop ORC. However, when the heat proportion of the second loop is 20% and 0%, increasing the second loop’s evaporating temperature, the power output, and thermal efficiency first rise and then decrease. When the heat distribution of the second loop is 0%, the series–parallel type changes into a series configuration. Thus, these results are similar to the series MLORC. It was evident that the series–parallel MLORC’s power output is lower than that of the series MLORC. Besides, it is observed that the more energy the first cycle recovers from the exhaust gas, the more power the system will output. This finding is consistent with that of Huang et al. [45]. Overall, these results suggest that, compared to the series MLORC, the series–parallel MLORC has worse performance. Therefore, optimization for the series–parallel MLORC is unnecessary, according to the analysis results.

### 5.5. Multi-Objective Optimization of MLORC

According to the analysis results, multi-loop ORCs with the parallel configuration and series configuration have better performance than the series–parallel configuration. Therefore, in this section, multi-objective optimizations are performed to compare the performance of the two types under the same number of cycles. Furthermore, MLORC with a different number of cycles is also compared under the same configuration.

In this study, there are four working fluids. Therefore, in each configuration, for dual-loop, the number of working fluid combinations is 16, for the triple-loop, the number of combinations is 64, and for quadruple-loop, the number of combinations is 256. Multi-objective optimization was carried out for each combination. After optimization, the TOPSIS method is performed to select the best individual in the Pareto Frontier of each combination. Then, GRA was used to rank these individuals. Under the same number of cycles, four combinations with the highest grey relation grade *R* were selected to compare the series and parallel configurations. With the same configuration, two combinations that have the highest *R* were picked out for comparison.

#### 5.5.1. MLORC with the Name Number of Loops in Different Configurations

**Figure 14:**
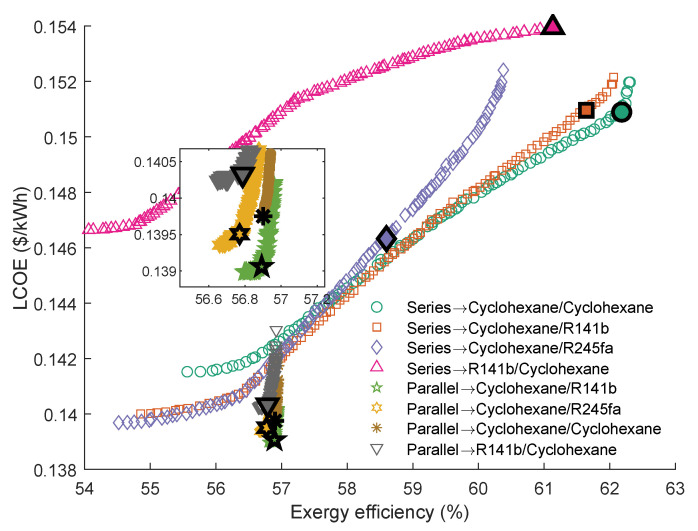
Pareto Frontiers of the series and parallel dual-loop ORC with the highest grey relation grade *R*.

Figure 14 shows the Pareto Frontiers of the series and parallel dual-loop ORC with the highest grey relation grade *R*. As can be seen, the series DLORC has a broader range of exergy efficiency than the parallel DLORC. Correspondingly, the DLORC in series has a broader range of the LCOE than in parallel configuration. The working fluid pair of cyclohexane and cyclohexane have the highest *R* in series DLORC. The fluid combination of cyclohexane and R141b performed best in parallel DLORC. Therefore, it is understood that the maximum exergy efficiency of the series DLORC is higher than that of the parallel DLORC. The maximum exergy efficiency of the series DLORC that uses cyclohexane\cyclohexane as working fluid is 62.31%, and the LCOE is 0.1520 $/kWh. The maximum $\eta_{\mathrm{exer}}$ of the parallel DLORC is 56.95%, which was obtained when cyclohexane\R141b were selected as the working fluids.

As can be seen from Table 10, cyclohexane\cyclohexane shows the best performance in series DLORC. Interestingly, cyclohexane\R245fa shows the third *R*. Although the exergy efficiency of this fluid pair is low, LCOE is also low.

Table 11 illustrates the parallel DLORC optimal parameters with the four highest *R*. It can be seen that the exhaust gas outlet temperature of the first cycle in parallel DLORC is 449.57 K. The results indicate that when the exhaust gas inlet temperature is 535.75 K, and the limit of the outlet temperature is 447.5 K, a single cycle is enough to recover the energy in the waste source. The results are consistent with analysis results shown in Figure 12.

**Figure 15:**
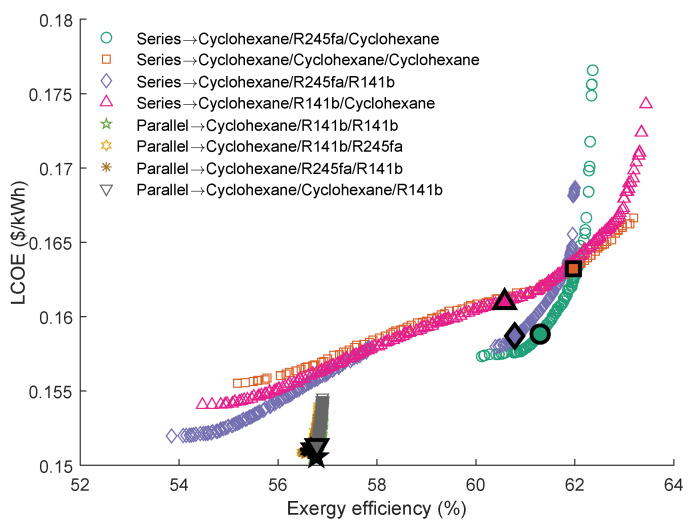
Pareto Frontiers of the series and parallel triple-loop ORC with the highest grey relation grade *R*.

The Pareto Frontiers of the series and parallel triple-loop ORC with the highest grey relation grade *R* are shown in Figure 15. It can be seen that the working fluid of the first cycle in parallel TLORC is cyclohexane. The results indicated that the Pareto Frontiers of the parallel TLORC are similar to that of the single ORC using cyclohexane as the working fluid. In series configuration, the cyclohexane\R245fa\cyclohexane has the highest *R*. The individuals in the Pareto Frontier of this working fluid pair are closer to the ideal solution. However, the exergy efficiency range of this Pareto Frontier is narrower than the other three. When using cyclohexane\R141b\cyclohexane, the series TLORC has the maximum exergy efficiency. Accordingly, the LCOE is a little larger than the other three. What is interesting in the Figure 15 is that, when using cyclohexane\R245fa\R141b, TLORC’s Pareto Frontier has a short interval. A possible explanation for this result is the PPTD limit in the intermediate heat exchanger. In series TLORC, there two heat exchangers, and the PPTD limit is 5 K. Therefore, for the entire system cycle, a temperature interval of 10 K cannot be used.

The series triple-loop ORC’ optimal parameters calculated by the TOPSIS method are presented in Table 12. As can be seen, that the evaporating temperature of the first cycle is almost equal. Furthermore, from the data in Table 12, it can be seen that the temperature difference between the evaporating temperature and condensation temperature of the second cycle is relatively small. These results revealed that series DLORC with suitable working fluids could fully utilize the energy in a single heat source.

**Figure 16:**
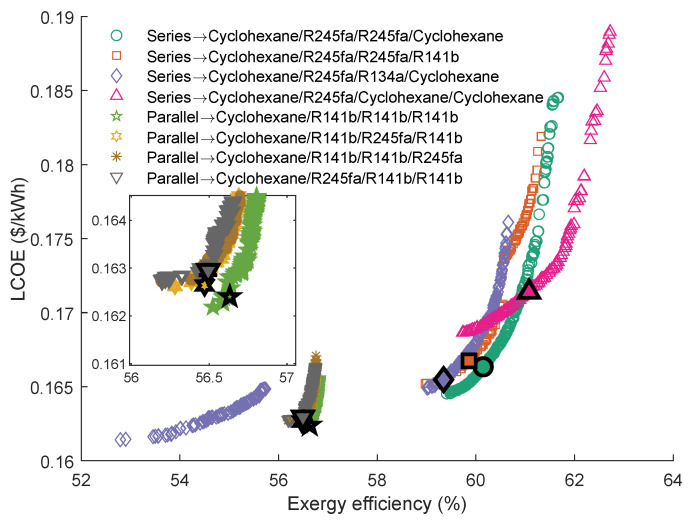
Pareto Frontiers of the series and parallel quadruple-loop ORC with the highest grey relation grade *R*.

Figure 16 provides the Pareto Frontiers of the series and parallel quadruple-loop ORC with the highest grey relation grade *R*. As can be seen, the Pareto Frontiers of the parallel QLORC are similar to that of the single ORC using cyclohexane as the working fluid. In series configuration, the cyclohexane\R245fa\R245fa\cyclohexane has the highest *R*. The exergy efficiency ranges of these four working fluid pairs are a little small. Furthermore, when using the cyclohexane\R245fa\cyclohexane\cyclohexane as working fluids, the series QLORC has the maximum exergy efficiency. Interestingly, in these four working fluid combinations, the fluid of the first cycle and the fluid of the second cycle in each combination are the same as the other combinations. In addition, as can be seen, two working fluid pairs have an interval on the Pareto Frontiers.

Table 13 presents the series quadruple-loop ORC’ optimal parameters calculated by the TOPSIS method. It is shown that the temperature differences between the evaporating temperature and condensation temperature of the second cycle and third cycle are slight. Thus, these results support the findings in Table 12.

#### 5.5.2. MLORC in Same Configuration with Various Number of Loops

**Figure 17:**
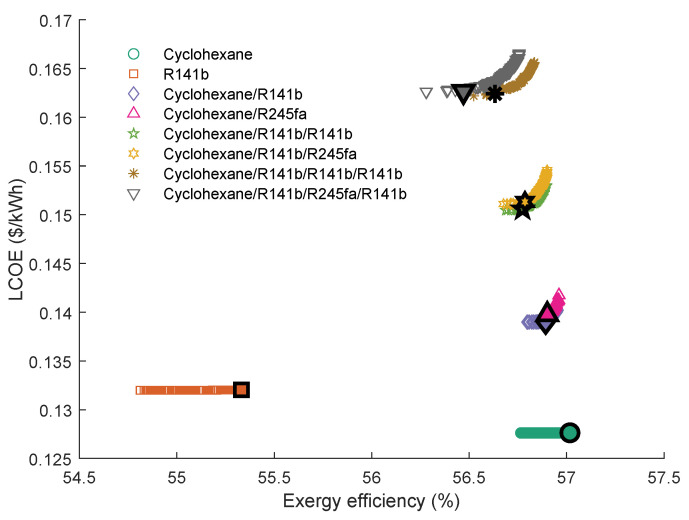
Pareto Frontiers of the parallel MLORC with different number of loops under the circumstance $T_{\mathrm{eg},\mathrm{in}}=537.75\;K,T_{\mathrm{eg},\mathrm{out}}=447.65\;K$.

Figure 17 presents the Pareto Frontiers of the parallel MLORC with various numbers of loops under the circumstance $T_{\mathrm{eg},\mathrm{in}}=537.75\;K,T_{\mathrm{eg},\mathrm{out}}=447.65\;K$. As can be seen, the single ORC using cyclohexane has the best performance. The result suggested that the single-cycle could be enough to recover the energy in the exhaust gas in a parallel configuration. Additionally, it is viewed that increasing the number of loops could cause an increase in LCOE. This result is consistent with the result in Figure 12.

**Figure 18:**
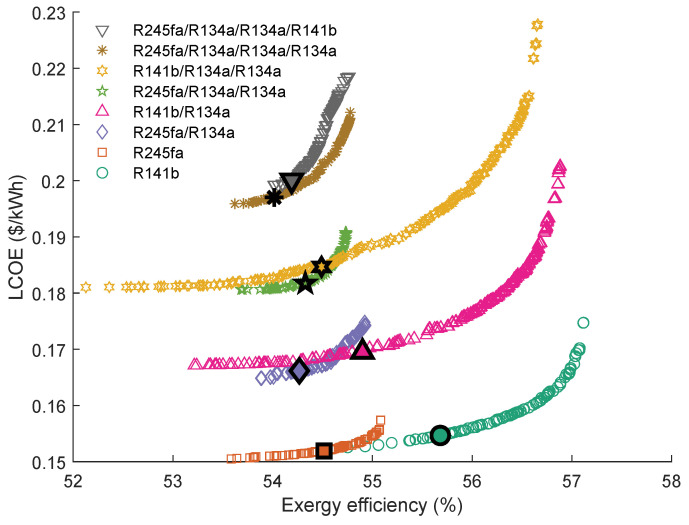
Pareto Frontiers of the parallel MLORC with different numbers of loops under the circumstance $T_{\mathrm{eg},\mathrm{in}}=450\;K,T_{\mathrm{eg},\mathrm{out}}=350\;K$.

Figure 18 illustrates the Pareto Frontiers of the parallel MLORC with various numbers of loops under the circumstance $T_{\mathrm{eg},\mathrm{in}}=450\;K,T_{\mathrm{eg},\mathrm{out}}=350\;K$. It is observed that the Pareto Frontier of the single ORC using R141b performed the best. There are some differences between this result and the result shown in Figure 12c. A possible reason may be that, in the result presented in Figure 12c, the working fluids were not optimal for the cycles. Interestingly, when R141b is used as the working fluid in the first cycle of the parallel MLORC, it has a wider exergy efficiency range than when R245fa is selected as the working fluid.

**Figure 19:**
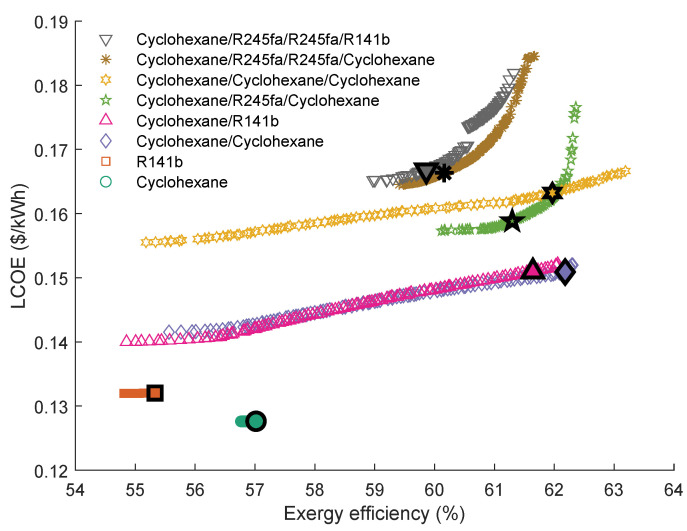
Pareto Frontiers of the series MLORC with different numbers of loops $T_{\mathrm{eg},\mathrm{in}}=537.75\;K,T_{\mathrm{eg},\mathrm{out}}=447.65\;K$.

Figure 19 shows that Pareto Frontiers of the series MLORC with various numbers of loops under the circumstance $T_{\mathrm{eg},\mathrm{in}}=537.75\;K,T_{\mathrm{eg},\mathrm{out}}=447.65\;K$. It can be seen that increasing the number of cycles can greatly increase the range of exergy efficiency. The series TLORC using cyclohexane\cyclohexane\cyclohexane may have the maximum exergy efficiency. However, the DLORC using cyclohexane\cyclohexane has the best performance. The reason may be that the economic indicator LCOE of the DLORC is much lower than that of the TLORC.

## 6. Conclusions

In summary, the thermoeconomic performance of Multi-Loop ORC under three various configurations was analyzed. Furthermore, multi-objective optimizations were conducted for the series and parallel MLORC using various working fluid combinations. The TOPSIS method was used to find the optimal solution on the Pareto Frontiers of each working fluid combination, and the GRA was applied to evaluate these solutions. Based on the analysis and optimizations, the conclusions can be drawn as follows:

1. For Multi-Loop ORC (MLORC) in series type, an optimal evaporation temperature for each loop allows the MLORC to maximize its power output and minimize the LCOE. Triple-loop ORC has the maximum power output. In this configuration, MLORC has an optimal number of cycles, which could maximize the power output. However, increasing the number of cycles inevitably leads to an increase in LCOE.
2. For dual-loop ORC in a parallel configuration, if the inlet temperature and outlet temperature of the exhaust gas are greater than the critical temperature of the working fluid, increasing the heat distribution of the second cycle would not affect the power output. If the inlet temperature is greater than the critical temperature, but the outlet temperature is less than the critical temperature, increasing the energy proportion of the second cycle will cause the power output to decrease first and then increase. If the inlet temperature and outlet temperature are both less than the critical temperature, increasing the heat distribution of the second cycle will cause the power to rise first and then drop.
3. For Series–Parallel Multi-Loop ORC, the more energy the first cycle absorbs from the exhaust gas, the higher the system power output.
4. Under the same number of loops, the series MLORC has a wider exergy efficiency range than the parallel MLORC. Moreover, the maximum exergy efficiency of the series MLORC is higher than the maximum exergy efficiency of parallel MLORC.
5. The series dual-loop ORC may be the suitable configuration for recovering energy from the exhaust gas. The optimal working fluids for this configuration are cyclohexane\cyclohexane. The exergy efficiency and LCOE of the series dual-loop ORC with the optimal parameters are 62.18% and 0.1509 $/kWh, respectively.

The present research provides guidelines in choosing the configuration for the Dual-Loop ORC or Multi-loop ORC. Based on this study, further work will be analyzing the effect of the temperature difference between the inlet and outlet of the heat source on Multi-Loop ORC performance and fluid selection.

## Figures and Tables

**Table 1:** The parameters of the exhaust gas [20].

Parameter	Unit	Value
Exhaust gas temperature (after turbine)	K	535.75
Exhaust gas mass flow (after turbine)	kg/s	10.33

**Table 2:** Composition of exhaust gas (%mol).

Parameter	Value
$O_{2}$	13.75
$N_{2}$	75.812
${\mathrm{CO}}_{2}$	4.703
$H_{2}O$	4.771
Ar	0.902
${\mathrm{SO}}_{2}$	0.062

**Table 3:** The thermodynamic properties of the four candidate working fluids.

Item	R134a [23]	R245fa [24]	R141b [10]	Cyclohexane [25]
Molar mass (kg/kmol)	102.03	134.05	116.95	84.16
Boiling temperature (K)	247.1	288.3	305.2	353.9
Critical temperature (K)	374.21	427.16	477.5	553.6
Critical pressure (kPa)	4059	3651	4210	4081
ODP	0	0	0.12	0
GWP	1430	1030	20	20

**Table 4:** Constants in the Kandlikar correlation.

Constant	Convective Region	Nucleate Boiling Region
$H_{1}$	1.1360	0.6683
$H_{2}$	−0.9	−0.2
$H_{3}$	667.2	1058.0
$H_{4}$	0.7	0.7
$H_{5}$ *	0.3	0.3

* $H_{5}=0$ for vertical tubes, and for horizontal tubes with $F\;r_{\mathrm{lo}}>0.04$.

**Table 5:** Coefficients in the module cost evaluation equations [20].

Equipment Type	$K_{1}$	$K_{2}$	$K_{3}$	$C_{1}$	$C_{2}$	$C_{3}$	$B_{1}$	$B_{2}$	$F_{M}$
Plate heat exchanger	4.6561	−0.2947	0.2207	0	0	0	0.96	1.21	1
Shell and tube heat exchanger	4.3247	−0.3030	0.1634	0.0381	−0.11272	0.08183	1.63	1.66	1.2
Condenser	4.6561	−0.2947	0.2207	0	0	0	0.96	1.21	1
Expander	2.2476	1.4965	−0.1618	0	0	0	/	/	3.8
Working pump	3.3892	0.0536	0.1538	−0.3935	0.3957	−0.00226	1.89	1.35	1.6

**Table 6:** Critical parameters of the NSGA II method.

Items	Value
Population size	100
Maximum iterations	100
Crossover probability	0.75 [35]
Mutation probability	0.25 [35]

**Table 7:** Main parameters of the thermodynamic model and decision boundaries.

Items	Value	Unit
Pump isentropic efficiency [40]	75	%
Expander isentropic efficiency [41]	80	%
Environment temperature [23]	25	°C
Exhaust gas outlet temperature	$\geq T_{\mathrm{dew}}$	°C
Seawater temperature	20	°C

**Table 8:** Comparison results of the thermodynamic model with the previous article [43].

Items	*W* (kW)	η (-)	$m_{r}$ (kg/s)	$P_{\mathrm{eva}}$ (kPa)	$T_{\mathrm{eva}}$ (K)	$P_{\mathrm{con}}$ (kPa)	$T_{\mathrm{con}}$ (K)
R134a [43]	147.5	0.0852	8.9667	3723.4	369.9	883.3	308
R134a (Present)	148.9	0.0853	8.967	3724.1	369.9	883.2	308
R11 [43]	290.3	0.166	7.487	3835.94	461	147.9	308
R11 (Present)	291.5	0.167	7.488	3837.4	461	147.9	308

**Table 9:** Comparison results of the economic model with the previous article [23].

Items	*W* (kW)$T_{\mathrm{ev}}$ (K)	$T_{\mathrm{con}}$ (K)	$T_{\sup}$ (K)	$T_{\mathrm{eg},\mathrm{out}}$ (K)	$T_{\mathrm{sw},\mathrm{out}}$ (K)	LCOE ($/kWh)	$\eta_{\mathrm{exer}}$ (%)
R717 [23]	296.32	284.20	1.54	297.89	282.78	0.341	28.17
R717 (Present)	296.32	284.20	1.54	297.89	282.78	0.332	28.25
R134a [23]	296.05	284.45	2.26	297.87	283.36	0.549	26.88
R134a (Present)	296.05	284.45	2.26	297.87	283.36	0.538	26.96

**Table 10:** The series dual-loop ORC’ optimal parameters calculated by the TOPSIS method.

Fluid Pairs	Cy\Cy	Cy\R141b	Cy\R245fa	R141b\Cy	Unit
$T_{\mathrm{ev},1}$	484.90	487.17	494.51	474.50	K
$T_{\sup,1}$	5.00	5.00	5.00	49.88	K
$T_{\mathrm{ex},\mathrm{out}}$	447.69	447.65	447.67	447.65	K
$T_{\mathrm{con},1}$	371.19	361.59	317.42	392.73	K
$T_{\mathrm{pp},1}$	5.08	5.04	5.00	5.00	K
$T_{\mathrm{con}}$	305.15	305.15	305.15	305.15	K
$T_{\mathrm{pp},\mathrm{con}}$	10.78	10.71	11.00	10.92	K
$\eta_{\mathrm{exer}}$	62.18	61.64	56.56	61.14	%
LCOE	0.1509	0.1510	0.1417	0.1540	$/kWh

**Table 11:** The parallel dual-loop ORC’s optimal parameters calculated by the TOPSIS method.

Fluid Pairs	Cy\R141b	Cy\Cy	Cy\R245fa	R141b\Cy	Unit
$T_{\mathrm{ev},1}$	496.69	496.68	520.67	473.60	K
$T_{\sup,1}$	5.00	5.00	5.00	49.13	K
$T_{\mathrm{ex},\mathrm{out}1}$	449.57	449.41	533.77	533.79	K
$T_{\mathrm{con},1}$	305.15	305.15	305.15	305.15	K
$T_{\mathrm{pp},\mathrm{con}1}$	10.98	11.00	5.01	5.03	K
$T_{\mathrm{ev},2}$	434.36	424.11	494.15	493.85	K
$T_{\sup,2}$	5.00	15.05	5.00	5.00	K
$T_{\mathrm{ex},\mathrm{out}2}$	447.65	447.65	447.65	447.65	K
$T_{\mathrm{con},2}$	305.15	305.15	305.15	305.15	K
$T_{\mathrm{pp},\mathrm{con}2}$	5.02	5.00	11.00	10.80	K
$T_{\mathrm{ex},\mathrm{limit}1}$	449.57	449.41	449.42	533.79	K
$\eta_{\mathrm{exer}}$	56.89	56.90	56.77	56.79	%
LCOE	0.1391	0.1398	0.1395	0.1403	$/kWh

**Table 12:** The series triple-loop ORC’ optimal parameters calculated by the TOPSIS method.

Fluid Pairs	Cy\R245fa\Cy	Cy\Cy\Cy	Cy\R245fa\R141b	Cy\R141b\Cy	Unit
$T_{\mathrm{ev},1}$	481.75	484.92	485.10	482.14	K
$T_{\sup,1}$	5.00	5.00	5.00	5.00	K
$T_{\mathrm{ex},\mathrm{out}}$	447.65	447.65	447.65	447.66	K
$T_{\mathrm{con},1}$	382.35	368.24	370.44	380.99	K
$T_{\mathrm{pp},1}$	5.00	5.00	5.00	5.00	K
$T_{\mathrm{con},2}$	372.57	362.17	362.97	364.91	K
$T_{\mathrm{pp},2}$	5.01	5.00	5.00	5.00	K
$T_{\mathrm{con}}$	305.15	305.15	305.15	305.15	K
$T_{\mathrm{pp},\mathrm{con}}$	11.00	11.00	11.00	10.96	K
$\eta_{\mathrm{exer}}$	61.30	60.41	60.60	61.97	%
LCOE	0.1588	0.1580	0.1581	0.1632	$/kWh

**Table 13:** The series quadruple-loop ORC’ optimal parameters calculated by the TOPSIS method.

Fluid pairs	Cy\R245fa\R245fa\Cy	Cy\R245fa\R245fa\R141b	Cy\R245fa\R134a\Cy	Cy\R245fa\Cy\Cy	Unit
$T_{\mathrm{ev},1}$	480.97	481.50	484.28	480.09	K
$T_{\sup,1}$	5.00	5.00	5.00	5.00	K
$T_{\mathrm{ex},\mathrm{out}}$	447.65	447.65	447.65	447.65	K
$T_{\mathrm{con},1}$	385.00	383.38	373.68	387.54	K
$T_{\mathrm{pp},1}$	5.01	5.04	5.07	5.00	K
$T_{\mathrm{con},2}$	376.20	374.65	364.70	380.84	K
$T_{\mathrm{pp},2}$	5.00	5.00	5.00	5.00	K
$T_{\mathrm{con},3}$	365.77	364.79	358.62	358.23	K
$T_{\mathrm{pp},3}$	5.00	5.04	5.00	5.00	K
$T_{\mathrm{con}}$	305.15	305.15	305.15	305.15	K
$T_{\mathrm{pp},\mathrm{con}}$	10.97	10.88	11.00	10.92	K
η−exer	60.16	59.86	59.35	61.08	%
LCOE	0.1663	0.1667	0.1655	0.1714	$/kWh

## Data Availability

Data is contained within the article.

## Abbreviations

The following abbreviations are used in this manuscript:

ORC	Organic Rankine Cycle
MLORC	Multi-Loop Organic Rankine Cycle
DLORC	Dual-Loop Organic Rankine Cycle
TLORC	Triple-Loop Organic Rankine Cycle
QLORC	Quadruple-Loop Organic Rankine Cycle
LCOE	Levelized cost of energy
CRF	Capital recovery factor
WHRS	Waste Heat Recovery System
PPTD	Pinch point temperature difference
NPO	Net power output
CEPCI	Chemical Engineering Plant Cost Index
LMTD	Logarithmic Mean Temperature Difference
TOPSIS	Technique for Order Preference by Similarity to Ideal Solution
NSGA II	Non-dominated Sorting Genetic Algorithm II
COM	Cost of operation
GRA	Grey Relational Analysis
MADM	Multiple Attribute Decision Making

## Symbols

The following symbols are used in this manuscript:

$\dot{Q}$	Heat transfer rate, kW
$c_{p}$	Specific heat, kJ/(kg K)
*W*	Power, kW
*N*	Number of loops
η	Efficiency, %
*T*	Temperature, K
$\dot{E}$	Exergy, kW
$\dot{I}$	Exergy destruction rate, kW
$\dot{m}$	Mass flow rate, kg/s
*s*	Entropy, kJ/(kg K)
*y*	Type of the ORC
*A*	Heat transfer area, $m^{2}$
$F_{P}$	Pressure factor
*U*	Total heat transfer coefficient, W/m${}^{2}$ K
*F*	Correction factor
*k*	Thermal conductivity, W/m${}^{2}$ K
*i*	Fouling resistance
δ	Thickness of tube or plate, m
α	Heat transfer coefficient, W/m${}^{2}$ K
*h*	Specific enthalpy, kJ/kg
Re	Reynolds number
$C_{\mathrm{BM}}$	Bare module equipment cost
$C_{p}$	Bare module cost for ambient pressure and carbon steel construction
μ	Viscosity, Pa·s
*g*	Acceleration of gravity, $m/s^{2}$
ρ	Density, kg/m${}^{3}$
γ	Latent heat, J/kg
*j*	Interest rate
Bo	Boling number
Pr	Prandtl number
*t*	time, h
$D_{e}$	Equivalent diameter, m
*K*	Contraction and expansion loss coefficients
Fr	Froude number
ξ	Relative closeness
LT	Life time
*x*	Mass fraction
*D*	Euclidean distances
ζ	Relational coefficient
*R*	Grey relational grade

## Subscripts

The following subscripts are used in this manuscript:

ev	Evaporator
r	Working fluid
eg	Gas
in	Inlet
out	Outlet
he	heat exchanger
ex	Expander
sw	Seawater
con	Condenser
pu	Pump
is	Isentropic
se	Series configuration
pa	Parallel configuration
sepa	Series–Parallel configuration
exer	Exergy
max	Maximum
min	Minimum
pl	Plate heat exchanger
st	Shell and tube heat exchanger
h	Hot side
c	Cold side
os	Outside
eq	Equivalent
ave	Average
l	Liquid
g	Gas
tp	Two-phase
sup	Superheat
dew	Dew point
pp	Pinch point
tot	Total
w	Wall
